# Prevalence of Physical Activity Among Adults in a Metropolitan Nigerian City: A Cross-Sectional Study

**DOI:** 10.2188/jea.JE20120116

**Published:** 2013-05-05

**Authors:** Adewale L Oyeyemi, Adetoyeje Y Oyeyemi, Zainab A Jidda, Fatima Babagana

**Affiliations:** Department of Physiotherapy, College of Medical Sciences, University of Maiduguri, Maiduguri, Borno State, Nigeria

**Keywords:** physical activity, sociodemographic characteristics, IPAQ, Nigerian adults

## Abstract

**Background:**

Baseline information on physical activity is relevant to controlling the epidemic of chronic noncommunicable diseases occurring in many African countries. However, standardized data on physical activity are lacking in Nigeria. We assessed the prevalence of physical activity and its relationships with sociodemographic characteristics in a subnational sample of Nigerian adults.

**Methods:**

A cross-sectional survey was conducted among a representative sample of 934 adults (age, 20–82 years) living in metropolitan Maiduguri, Nigeria. Physical activity was measured using the validated Nigerian version of the International Physical Activity Questionnaire (Hausa IPAQ-SF). Using the World Health Organization (WHO) guideline, participants were classified as sufficiently active or insufficiently active. Sociodemographic correlates of sufficient physical activity were identified using multinomial logistic regression.

**Results:**

Overall, 68.6% of Nigerian adults were sufficiently active. There was no significant difference (*P* > 0.05) in prevalence of physical activity between men (68.0%) and women (69.3%), but physical activity tended to decrease with increasing age category, especially among men. Physical activity prevalence was positively associated with being married (OR = 1.52, CI = 1.04–4.37) and blue collar work (OR = 2.19, CI = 1.16–4.12) and negatively associated with car ownership (OR = 0.38, CI = 0.17–0.86) and higher income (OR = 0.54, CI = 0.10–0.95).

**Conclusions:**

The prevalence of physical activity varied between sociodemographic subgroups of Nigerian adults; thus, public health policies and interventions based on ecologic models of health behaviors may be warranted in promoting physical activity in Nigeria.

## INTRODUCTION

Physical activity is an important determinant of health and is associated with reduced risk of chronic diseases such as cardiovascular disease (CVD), diabetes, obesity, and certain form of cancers.^1^ About 2 million deaths per year are attributable to physical inactivity worldwide, and more than 80% of deaths from chronic diseases occur in developing countries.^1^^,^^2^ The nutritional, lifestyle, and socioeconomic transitions occurring in many developing countries have significantly increased the burden of chronic noncommunicable diseases (NCDs) in the region (including in Nigeria and other African countries).^1^^,^^3^ In addition, rapid urbanization and its attendant unhealthy dietary habits and reduced physical activity have been linked to the epidemiologic transition from infectious communicable diseases to chronic NCDs in the African continent.^4^

Data on physical activity in developing African countries are needed in order to effectively formulate policies and programs that prevent chronic conditions and NCDs.^5^ Recently, comparable data on physical activity prevalence in many African countries, excluding Nigeria, were published.^5^^,^^6^ The prevalence rates of physical activity in the studied African countries varied widely, although the reasons for these variations were not obvious. For example, the prevalence of physical inactivity was reported to be 52.6%, 49.1%, and 44.7% in Mauritania, Swaziland, and South Africa, respectively, but was as low as 7.8%, 8.4%, and 8.8% in Burkina Faso, Malawi, and Ghana, respectively.^6^ Similarly, Mozambique and Malawi, in southeastern Africa, had the highest reported prevalence of physical activity (around 95%), whereas Mali and Mauritania, 2 sub-Saharan West African countries, were reported to have a prevalence of about 50%.^5^ Examining country-specific population-based data on physical activity prevalence may be necessary to identify promising interventions that are appropriate to the local environment, especially in Nigeria, where such data are scarce.

With a population of more than 140 million,^7^ Nigeria is the most populous country in Africa, and physical inactivity-related NCDs are reported to be increasing.^4^^,^^8^ Thus, the need for baseline national or subnational data on physical activity for surveillance is urgent. Such information could help to identify populations and subgroups at risk of physical inactivity-related morbidity and mortality in Nigeria. Empirical data on physical activity prevalence could be used by governments to formulate policies and programs aimed at controlling the increase in NCDs.^9^^,^^10^ In this study, we assessed the prevalence and sociodemographic correlates of physical activity in a subnational sample of adults in Maiduguri, Nigeria.

## METHODS

### Setting

Maiduguri is the largest city and capital of Borno State, in northeastern Nigeria. It has an estimated population of 749 123, and consists of Kanuri, Shuwa Arabs, Hausa, Fulani, and other ethnic groups.^7^^,^^11^ Borno State covers an area of 72 609 square kilometers (population density, 57 people/square kilometer) and attracts immigrants from the Republic of Cameroon, Republic of Niger, and Republic of Chad.^7^^,^^12^ The diverse inhabitants of Maiduguri predominantly use the Hausa language as a common means of communication and commercial activities.^11^ The Figure shows a map indicating the location of Maiduguri.

**Figure.**
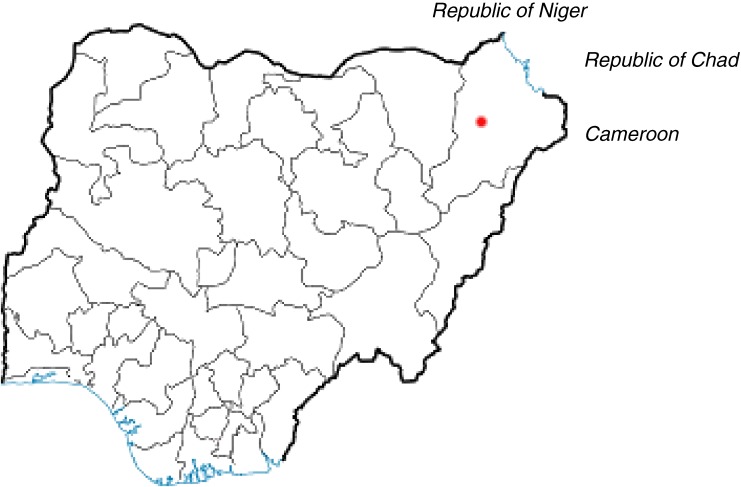
Map of Nigeria showing city of Maiduguri. ● Maiduguri, Nigeria.

### Participants and design

Multistage probability cluster sampling was used to recruit 934 adults from different households in Maiduguri Metropolitan Council. Using the international guidelines for conducting community surveys,^13^ 7 of the 15 political wards into which Maiduguri Metropolitan Council is divided were randomly selected by lottery (stage 1). In each of the 7 selected wards, 2 enumeration areas per ward were randomly selected, for a total of 14 enumeration areas (stage 2). For each of the selected enumeration areas, houses were numbered on-site, and every odd-numbered house was approached, to determine study interest and eligibility (stage 3). Participants were invited to participate in the study if they met the following eligibility criteria: (1) residence in the identified household, (2) age older than 19 years, (3) absence of any disability that prevented walking, and (4) ability to complete or understand a survey in the English or Hausa language.

Sample size was calculated using Daniel’s formula for prevalence studies.^14^ Using an effect size [d] of 0.05 and a design effect of 3 (based on sampling),^15^ we determined that 1152 participants were needed to estimate the population prevalence of physical activity with good precision.^14^ A total of 1400 adults (100 per enumeration area) were contacted for the study in a door-to-door house survey, but only 934 (age range, 20–84 years) consented and provided a complete and usable survey, yielding a response rate of 66.7% (934/1400). A cross-sectional survey design was used to collect data between March and May 2011. The study questionnaire was administered by interview, and the English language was used for participants who did not understand Hausa. All participants provided informed consent, and the study was approved by the Human Research Ethics Committee of the University of Maiduguri Teaching Hospital, Nigeria (ADM/TH/EC/75; 21/01/2011).

### Physical activity measure

Information on physical activity behaviors was collected with the Nigerian version of the International Physical Activity Questionnaire-Short Form (Hausa IPAQ-SF). A detailed description of the development of the Nigerian version of the IPAQ-SF is available elsewhere.^16^ Briefly, the original English IPAQ-SF was culturally adapted by a panel of experts, translated into Hausa by 2 independent translators, synthesized by a panel of 6 experts (including the translators), back-translated by another 2 translators, and subsequently subjected to expert committee review and pretesting. The final product, the Hausa IPAQ-SF, is available in both Hausa and English (eQuestionnaire). The English edition of the Hausa IPAQ-SF was used for participants (*n* = 63) in the present study who did not understand Hausa. The Hausa IPAQ-SF included 7 items, estimated the time spent being physically active in the last 7 days, and measured vigorous-intensity activities, moderate-intensity activities (not including walking), walking, and sitting time. These activity categories can be treated separately to obtain the activity pattern or may be multiplied by their estimated intensity in METs and summed to gain an overall estimate of PA in a week (www.ipaq.ki.se). One MET represents the energy expended while sitting quietly at rest and is equivalent to 3.5 ml/kg/min of VO_2_.^17^ The MET intensities used to score IPAQ in this study were vigorous (8 METs), moderate (4 METs), and walking (3.3 METs) (www.ipaq.ki.se).

Physical activity levels were initially classified as low, moderate, or high intensity, defined by the IPAQ core group (http://www.ipaq.ki.se) as follows: Low—no activity or some activity reported, but not enough to satisfy the requirements of the other activity categories; Moderate—any of the following 3 criteria: (a) 3 or more days of vigorous-intensity activity for at least 20 minutes per day, (b) 5 or more days of moderate-intensity activity or waking for at least 30 minutes per day, or (c) 5 or more days of any combination of walking, moderate-intensity, or vigorous-intensity activities achieving a minimum of 600 MET-minutes per week; High—either of the following 2 criteria: (a) 3 or more days of vigorous-intensity activity accumulating at least 1500 MET-minutes per week or (b) 7 days of any combination of walking or moderate- or vigorous-intensity activities achieving a minimum of 3000 MET-minutes per week.

These 3 groups were then categorized as sufficiently physically active or physically inactive. The sufficiently physically active group included participants in the moderate- or high-intensity categories who met the WHO physical activity recommendation. According to the new WHO global standard, satisfying the recommendations for healthy physical activity was defined as engaging in at least 150 minutes of moderate-intensity activity per week, 75 minutes of vigorous-intensity activity per week, or an equivalent combination of moderate- and vigorous-intensity activity.^18^ The test–retest reliability (ICC = 0.33–0.73) and concurrent validity (ρ = 0.78–0.92) of the Hausa version of the IPAQ-SF in Nigeria were found to be good.^16^ Acceptable test–retest reliability (*r* = 0.70–0.97) and criterion validity (*r* = 0.23), as compared with accelerometer monitoring, has been reported for the IPAQ in both developed and developing countries.^19^

### Sociodemographic characteristics

Sociodemographic information on age, sex, ethnic group, marital status, household motor vehicle ownership, income, education level, and occupation was elicited from participants. Participant age was grouped into 5 categories: 20 to 34 years, 35 to 44 years, 45 to 54 years, and 55 years or older. Marital status was classified as single/never married, married, and divorced/separated. Education level was classified as more than secondary school education, secondary school education, and less than secondary school education. Occupation was categorized into 3 groups: white collar (office worker), blue collar (artisan, trader, farmer, etc.), and not employed (homemaker, student, retired, or unable to work). Participant income was classified into 4 groups: less than 15 000, 16 000 to 45 000, 46 000 to 90 000 and greater than 90 000 naira/month (15 000 naira is approximately 100 US dollars). Tribe was classified as Hausa/Fulani, Kanuri/Shuwa, Yoruba, Ibo, or other, and household motor vehicle ownership was classified as yes or no.

### Statistical analyses

The cluster sampling design was taken into account during analyses by using information on primary sampling units (PSUs), sampling weights for participants, and finite population correction. The complex sample survey capabilities of SPSS were used to analyze the data, after specifying that there were 7 strata and that the PSUs in these strata were households.^20^^,^^21^ The sampling weights used to adjust for probability of selection during sampling were computed using the process described by Lameshow and colleagues.^21^ All statistical analyses were performed using SPSS software version 15.0 (SPSS Inc., Chicago, IL, USA).

Estimates of the prevalence of physical activity and other categorical variables were reported as proportions with 95% CIs. Continuous variables such as age were reported as mean (SEM). The χ^2^ test was used to compare physically active and inactive participants relative to sociodemographic characteristics, and the significance level was defined as a *P* value less than 0.05. Multinomial logistic regression analysis with adjusted odd ratios (ORs) and 95% CIs was used to assess associations of physical activity with sociodemographic variables. ORs with 95% CIs were calculated against the reference category of participants aged 20 to 34 years, those who were single/never married, those with more than a secondary school education (higher education), those in a white collar occupation, those without a car in their household, and those earning less than 15 000 naira/month. Adjustment was made for all studied variables. We did not analyze multinomial logistic models separately for each sex, due to the relatively small sample size and to avoid a suppression effect.

## RESULTS

Table 1 shows the sociodemographic characteristics and prevalence of physical activity and inactivity among participants. The sample comprised 395 (42.3%) women and 539 (57.7%) men with a mean (SEM) age of 38.1 (1.7) years. Prevalence of physical activity was 68.6%, and about one-third of participants (31.4%) were physically inactive. Participants with less than a secondary school education were significantly more physically active (76.7%) than those with a secondary school education (66.5%) and those with more than a secondary school education (60.9%). Physical activity was significantly greater among those who had blue collar jobs (artisan, trader) than among those with white collar jobs and those who were not employed (homemaker, student, retired, or unable to work). Participants who did not own a car (77.9%) were significantly more physically active than those who did (57.6%). The highest prevalence rates of physical activity were among participants who were divorced/separated (80.4%), those without a car (77.4%), those with a blue collar job (76.6%), those with less than a secondary education (76.6%), and those with the lowest income (72.6%).

**Table 1.** Prevalence of physical activity overall, by participant sociodemographic characteristics (*n* = 934)

	*n*^a^ (%)	*n*^a^ = 640	*n*^a^ = 294	*P* value^b^

		% physical activity (95% CI)	% physical inactivity (95% CI)	
All	934 (100.0)	68.6 (46.6–84.5)	31.4 (15.5–53.4)	
Sex				0.753
Women	394 (42.1)	69.3 (48.9–84.1)	30.7 (15.9–51.1)	
Men	540 (57.9)	68.0 (44.1–85.2)	32.0 (14.8–55.9)	
Age group				0.735
20–34 years	441 (47.2)	72.4 (33.6–94.9)	27.6 (5.1–66.4)	
35–44 years	240 (25.7)	72.6 (40.7–92.1)	27.4 (7.9–59.3)	
45–54 years	140 (15.0)	69.8 (13.4–97.2)	30.2 (2.8–86.6)	
≥55 years	113 (12.1)	60.9 (34.2–82.3)	39.1 (17.7–65.8)	
Marital status				0.078
Single	189 (20.3)	59.6 (29.8–83.6)	40.4 (16.4–70.2)	
Married	666 (71.3)	69.7 (46.5–85.9)	30.3 (14.1–53.5)	
Divorced	79 (8.4)	80.4 (48.2–94.7)	19.6 (5.3–51.8)	
Ethnic group				0.352
Hausa/Fulani	348 (37.2)	70.4 (47.8–86.3)	29.6 (13.7–52.6)	
Kanui/Shuwa	254 (27.2)	72.0 (53.9–85.0)	28.0 (15.0–46.1)	
Igbo	64 (6.9)	67.0 (40.9–85.7)	33.0 (14.3–59.1)	
Yoruba	114 (12.2)	57.1 (37.9–74.4)	42.9 (25.6–62.1)	
Others	155 (16.6)	67.7 (39.9–87.3)	32.3 (12.7–61.0)	
Education level				0.046*
>Secondary	267 (28.7)	60.9 (32.8–79.7)	39.1 (20.3–61.8)	
Secondary	333 (35.8)	66.5 (44.9–82.9)	33.5 (17.1–55.1)	
<Secondary	330 (35.5)	76.6 (50.1–91.4)	23.4 (8.6–49.9)	
Occupation				0.038*
White collar	151 (16.2)	60.0 (41.7–75.8)	40.0 (24.2–58.3)	
Blue collar	451 (48.4)	76.6 (52.2–90.8)	23.4 (9.2–47.8)	
Unemployed	329 (35.4)	61.5 (32.7–84.0)	38.5 (16.0–67.3)	
Car ownership				0.025*
No	508 (54.5)	77.9 (51.7–92.1)	22.1 (7.9–48.3)	
Yes	425 (45.5)	57.6 (36.5–76.1)	42.4 (23.9–63.5)	
Income (naira)^c^				0.120
<15 000	363 (40.9)	73.6 (50.2–88.5)	26.4 (11.5–49.8)	
16 000–45 000	360 (40.6)	72.2 (50.6–86.8)	27.8 (13.2–49.2)	
46 000–90 000	86 (9.7)	58.2 (36.7–77.0)	41.8 (23.0–63.3)	
>90 000	78 (8.8)	60.0 (21.8–89.0)	40.0 (11.0–78.2)	

^a^Values based on estimated population size (values may not sum to 100.0, due to rounding).

^b^*P* value based on χ^2^ test; *P < 0.05 considered significant.

^c^Classification based on Nigerian salary scale.

Table 2 shows sex differences in physical activity prevalence among physically active participants across different age groups, occupations, income groups, educational levels, and car ownership status. More men than women were physically active, and the proportion of physically active individuals tended to decrease with increasing age among men and increase with increasing age among women. While the proportion of physically active men decreased from 75.6% in the youngest group to 53.6% in the oldest group, the proportion of physically active women increased from 68.1% in the youngest group to 69.5% in the oldest group. There were no clear sex differences in the patterns of physical activity prevalence among physically active adults in relation to income group, education level, or car ownership. While the prevalence of physical activity tended to increase with higher education among both women (from 58.8% to 76.0%) and men (62.0% to 77.1%), prevalence tended to decrease with higher income (from 75.0% to 54.1% among women and from 71.7% to 62.4% among men) and with car ownership (from 79.8% to 51.9% among women and from 76.1% to 60.6% among men) in both sexes.

**Table 2.** Patterns of physical activity prevalence by sociodemographic characteristics among physically active men and women (*n* = 640)

	*n*^a^	Women^b^ (*n* = 272)	Men^b^ (*n* = 368)
	
		% physical activity (95% CI)	% physical activity (95% CI)
Age group			
20–34 years	231	68.1 (25.9–90.8)	75.6 (39.8–94.2)
35–44 years	206	75.8 (43.8–93.5)	68.9 (34.8–91.8)
45–54 years	124	76.4 (15.6–98.1)	65.1 (12.2–97.0)
≥55 years	79	69.5 (36.4–88.1)	53.6 (31.9–82.8)
All age groups	640	69.3 (48.9–84.1)	68.0 (44.1–85.2)
Education level			
>Secondary	163	58.8 (39.0–76.2)	62.0 (37.3–81.8)
Secondary	223	67.7 (47.5–82.9)	65.8 (40.9–84.2)
<Secondary	253	76.0 (53.7–89.6)	77.1 (43.8–93.6)
Occupation			
White collar	92	64.6 (51.8–75.6)	58.0 (37.0–76.5)
Blue collar	346	75.2 (43.6–92.2)	77.3 (55.4–90.3)
Unemployed	202	66.5 (47.9–81.1)	52.7 (10.2–91.6)
Car ownership			
No	396	79.8 (51.7–89.4)	76.1 (50.2–91.0)
Yes	244	51.9 (34.8–68.5)	60.6 (36.2–80.6)
Income (naira)^c^			
<15 000	267	75.0 (51.7–89.4)	71.7 (45.3–88.6)
16 000–45 000	260	72.2 (51.7–86.3)	72.2 (49.5–87.3)
46 000–90 000	50	65.9 (43.4–83.0)	55.9 (30.4–78.6)
>90 000	47	54.1 (22.1–89.0)	62.4 (21.7–90.9)

^a^Values based on estimated population size (values may not sum to 640 due to missing data).

^b^Data are from physically active participants only (*n* = 640), not the overall study sample.

^c^Classification based on Nigerian salary scale.

Table 3 shows the ORs, SEMs, and CIs for the association between prevalence of physical activity and sociodemographic variables. Married participants were about 52% more likely to be physically active (OR = 1.52, CI = 1.02–4.73) than those who were single or never married. Participants with blue collar jobs were more than twice as likely to be physically active (OR = 2.19, CI = 1.16–4.12) as those who had white collar occupations or were unemployed. On the other hand, participants were less likely to be physically active if they owned a car (OR = 0.38, CI = 0.17–0.86) or had a monthly income greater than 90 000 naira (OR = 0.54, CI = 0.10–0.95).

**Table 3.** Associations between sociodemographic variables and sufficient physical activity (*n* = 934)

Variables	Physical activity		

	OR^a^	SE (OR)	95% CI
Sex			
Male	1.00		
Female	1.06	0.171	0.69–1.61
Age group (years)			
20–34	1.00		
35–44	1.97	1.258	0.31–12.57
45–54	1.83	1.328	0.25–13.48
≥55	1.49	0.799	0.10–32.26
Marital status			
Single/never married	1.00		
Married	1.52	0.379	1.04–4.37*
Divorced/separated	2.56	0.427	0.39–18.64
Education level			
>Secondary education	1.00		
Secondary education	1.28	0.416	0.75–2.19
<Secondary education	2.10	0.231	0.76–5.79
Occupation			
White collar	1.00		
Blue collar	2.19	0.393	1.16–4.12*
Unemployed	1.06	0.484	0.41–2.78
Car ownership			
No	1.00		
Yes	0.38	0.328	0.17–0.86*
Income (naira)			
<15 000	1.00		
16 000–45 000	0.93	0.696	0.55–1.59
46 000–90 000	0.50	0.625	0.19–1.06
>90 000	0.54	0.391	0.10–0.95*

OR: odds ratios, SE: standard error.

^a^Odds ratios adjusted for all variables in table; *significant odds ratio.

## DISCUSSION

To our knowledge, no published study has assessed the prevalence of physical activity and the way in which sociodemographic factors relate to physical activity levels in a subnational sample of Nigerian adults. This study revealed that about 68% of participants met WHO recommendations for sufficient physical activity. This prevalence is lower than those reported in 18 of the 22 African countries that participated in the recent WHO Stepwise approach to chronic disease risk factor surveillance.^5^ Only in Ivory Coast (66.7%), Algeria (66.5%), Mauritania (53.1%), and Mali (46.8%) was the prevalence of physical activity lower than in the present study. However, our finding appears to reflect regional patterns of physical activity in Africa as observed in the WHO African study. In that study,^5^ most countries located in the same sub-region of West Africa as Nigeria had physical activity prevalence similar to that in the present study.

The prevalence rate of physical activity in Nigeria, as in other West African countries, appears to be lower than that of countries in other regions of Africa. However, we advise caution in directly comparing our results with those from the study of 22 African countries, because although the same cut-off was used to determine prevalence of physical activity, different measures were used in the studies. The IPAQ used in the present study was designed for population surveillance of physical activity^19^ but has been reported to overestimate physical activity prevalence as compared with the Global Physical Activity Questionnaire (GPAQ) used in the study of 22 African countries^5^ and other measures of physical activity.^22^^–^^24^ However, the GPAQ was not used in the present study because, unlike the IPAQ,^16^ it has not been validated and tested in Nigeria. Nevertheless, our findings provide preliminary insight into physical activity prevalence among Nigerian adults.

Although, no significant difference was found in the prevalence of physical activity between men and women, physical activity tended to decrease with increasing age among active men but tended to increase with increasing age among active women. This finding is contrary to those from some other countries, where physical activity level was reported to be higher among men than among women and prevalence rates tended to decrease with increasing age in both sexes.^6^^,^^25^^–^^32^ While it is difficult to explain these discrepant findings, our results may reflect the very high level of physical activity among divorced/separated (80.4%) adults, which mostly comprised older women. Perhaps Nigerian women who were divorced/separated and thus older had more work responsibilities, in addition to their traditional domestic duties (house/yard chores). If so, they would have had more job-related physical activity as compared with married or single women, who were younger. Also, older women in this study had lower incomes and education level and were less likely to own a car. The routine of walking daily to the market or doing shopping among these older women might explain why physical activity increased with increasing age among physically active Nigerian women in this study.

Being married was positively associated with sufficient physical activity. This is contrary to findings from previous international studies, which found positive associations between physical activity and being single.^25^^,^^26^ Because guidelines for defining sufficient physical activity in the international literature are mostly based on leisure time activity,^17^^,^^18^ it is possible that married participants in the present study were more physically active in other domains of physical activity, such as work- and transport-related physical activity, than in recreational physical activity. Married African adults, especially men, may engage in more job- and transport-related physical activities because of societal expectations that they be breadwinners of the family. However, information on parity was not included in our study, and thus it is unclear whether being married per se or having children was responsible for the present discrepant result. Future research should fully explore the effect of marital status on physical activity behaviors of African adults.

We found that participants with high incomes (the high socioeconomic group) were less likely to be active than those with lower incomes. Individuals with high socioeconomic status may have jobs that are less physically demanding; thus, their overall physical activity would be lower than those with lower incomes, whose jobs might be more physically demanding. Our finding that participants with blue collar jobs (traders, artisans, farmers, etc.) were twice as likely to be physically active than those with white collar jobs (office workers) confirms this hypothesis. High socioeconomic groups in Nigeria tend to be senior civil servants, bureaucrats, technocrats, professionals and business executives. In developed countries, the leisure and vacation activities of individuals of high socioeconomic status tend to include sports and games; however, for Nigerians of high socioeconomic status, leisure and vacation tend to be passive, with little physical activity. Furthermore, appreciation of wellness and health promotion activities is yet to permeate society in this part of the world.

Despite the importance of active commuting (cycling and walking) as key means of energy expenditure and its resulting contribution to health,^33^^–^^36^ automobiles are gradually becoming the dominant form of transportation in most countries, severely reducing active modes of transportation.^37^ The likelihood of walking or cycling is believed to be inversely related to the number of automobiles per household, regardless of income level.^33^ Similarly, about 61% of adults who own a car (motorized transportation/passive commuting) in the present study were not sufficiently physically active to meet WHO recommendations and were less likely to be active as compared with their counterparts who did not own a car. These findings have implications for physical activity intervention in Nigeria, because household ownership of a car can easily be misconstrued as economic progress and an improved standard of living in African society. Because of the rapid urbanization and Westernization taking place in many African countries, it may be appropriate to advocate for integrated preventive strategies that focus on positive behavioral change. Such strategies could be based on ecologic models of behavior, which acknowledge that health behaviors are influenced by multiple levels, including individual, social/cultural, built environmental, and policy dimensions.^38^ Interventions based on ecologic models can affect entire populations on a relatively permanent basis^39^ and are the dominant approach for physical activity promotion worldwide.^9^^,^^40^

This study has some limitations that should be considered when interpreting the results. Although the sample was selected to be representative, the response rate was comparatively low, which could compromise the external validity and generalizability of the findings. The use of a self-report measure of physical activity, with the potential for information bias, is another limitation of this study. Overreporting due to social desirability leading to overestimation of the prevalence of physical activity has been reported for the IPAQ.^22^ Moreover, some of the participants in the present study were older than 69 years, the upper age limit for which the IPAQ was designed. It is not clear if the IPAQ can adequately capture physical activity level for older age groups; however, previous studies have used the IPAQ to assess physical activity among adults older than 69 years,^22^^,^^26^^,^^41^ and problems associated with the IPAQ were found not to be age-related.^42^ Also, the validity of the short version of the IPAQ in adequately capturing patterns of physical activity has been challenged.^43^ The short IPAQ does not differentiate between activity contexts. For example, individuals may engage in walking activity solely for leisure or transportation or engage in physical activity as part of house chores, sports, or for job commuting or transportation purposes. A means to address this shortcoming is the use of domain-specific measure such as the Global Physical Activity Questionnaire (GPAQ), which was developed using a process similar to that for the IPAQ.^44^ However, there are similar concerns with respect to GPAQ use, namely, its potential for measurement error and its comparability among countries.^45^ However, a culturally adapted version of the IPAQ long-form may be a much better alternative for future studies in the African continent, because the leisure and transport components of the IPAQ long-form seem to be the most relevant for population surveillance and public health intervention.^46^

Despite the above limitations, in view of its standardized survey methodology and measures and appropriate statistical methods, this study provides a valuable snapshot of physical activity prevalence and its patterns among Nigerian adults with different sociodemographic characteristics. The findings have implications for identifying the sociodemographic groups in Nigerian society that need to be targeted for effective interventions promoting physical activity.

### Conclusion

The present study is one of the first to use WHO recommendations and guidelines to estimate the prevalence of physical activity among Nigerian adults. About 68% of Nigerian adults living in Maiduguri city met the WHO recommendation. However, the proportion of adults that met these recommendations for physical activity varied significantly by sociodemographic characteristics: those who were divorced/separated, did not own a car, and had a lower socioeconomic status—as indicated by low income, low education level, and blue collar occupation—were more likely to be physically active. Given that the prevalence of physical activity varied between sociodemographic subgroups, interventions based on ecologic models of health behaviors may be necessary in promoting physical activity among Nigerian adults.

## ONLINE ONLY MATERIALS

eQuestionnaire. International Physical Activity Questionnaire, Short Last 7 Day Version.
